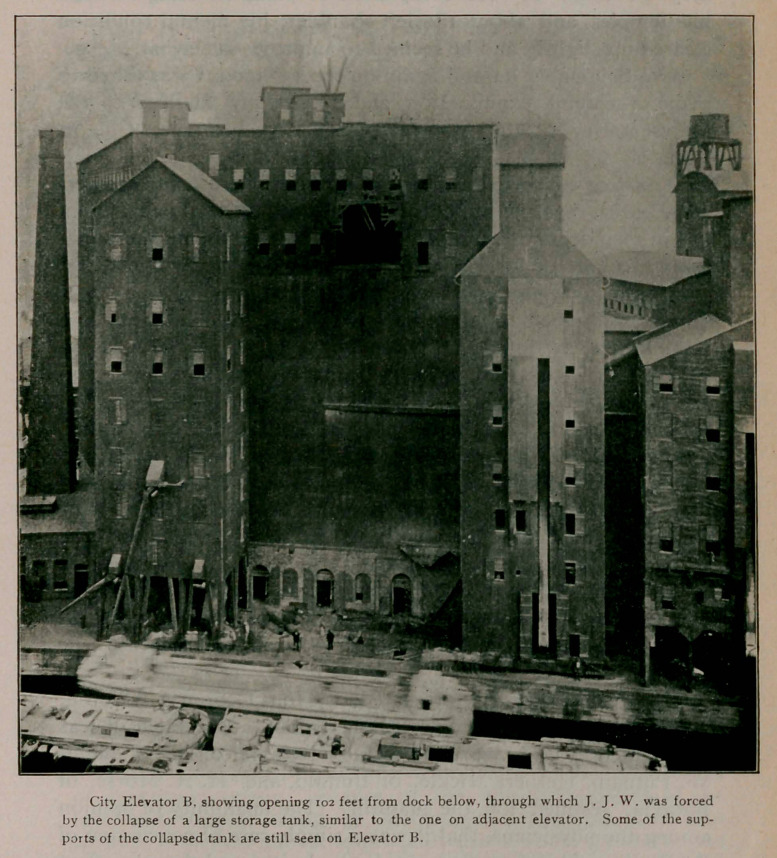# Report of Two Interesting Medico-Legal Cases1Read at the 35th annual meeting of the Medical Association of Central New York, held at Syracuse, N. Y., October 21, 1902.

**Published:** 1903-06

**Authors:** William C. Krauss

**Affiliations:** Buffalo, N. Y.; 479 Delaware Avenue


### Report of Two Interesting Medico-legal Cases.[1]

By WILLIAM C. KRAUSS, M. D., Buffalo, N. Y.

THE two following medico-legal cases which came under my observation within the past year, offer peculiarities which make this publication desirable. They show—the one how slight a shock or injury may produce serious or fatal results; the other, how great or incredible a fall and yet no fatal or serious results followed. Both cases are therefore remarkable and teach the lesson that physical injury is not commensurate with the degree of violence sustained.

J. J. W., aged 63; height, 5 feet 9 inches; weight, 150 pounds; occupation, worked in an elevator for about forty-two years; constitution, unusually strong and robust; had never been sick or disabled, and not addicted to liquor or tobacco.

For the past fifteen years he has been employed at the City Elevator B, located at the corner of Michigan and Ohio Streets, Buffalo, N. Y. This elevator, owned by the Western Transit Company, was a grain storage elevator, built of wood with sheet iron siding, about 140 feet in height from the dock to the roof.

1. Read at the 35th annual meeting of the Medical Association of Central New York, held at Syracuse, N. Y., October 21, 1902.

On the roof, supported by a frame work of timber, was a water tank with a capacity of 9,000 gallons. This tank was generally supposed to be filled with water ready for emergency and was connected with the elevator proper by a small size iron water pipe.

On the evening of August 7, 1900, Mr. W., after helping unload one of the company's grain boats, went to his office on the machinery floor of the elevator, which was located on the next to the top floor and directly under the water tank. The supports of this tank had been superficially examined some time before, and some of the timber found defective and repairs had been partially made.

On the night of August 7, 1900, without any warning, the supports gave way, the tank crashed through the roof and upper floor and burst into the room occupied by Mr. W., tearing a large hole out of the side of the elevator, carrying him to the dock below, a distance of 102 feet. He was picked up in an unconscious condition and taken to the Fitch Emergency Hospital. A careful examination by the hospital staff failed to find any fractures or dislocations, and no marked abrasions or tumefactions anywhere about the body.

His family physician, Dr. Herbert Mickle, was called and his report taken from the testimony at the trial is as follows:

I was called on August 7, 1900, to see him at the Emergency Hospital, where he had been taken after a fall sustained that morning. I found him in bed in an apparently semi-conscious condition, suffering severely from shock, cold sweat, cold surface of the body and general depression. At the request of the family and at my own suggestion, he was moved to his home, or his son's home, on Herkimer Street, in an ambulance, and I took charge of the case and attended him there until the following December, 1900. His condition at first was one of apparently only semi-consciousness; he would respond to questions, but with hesitation; there was a condition of mental semi-consciousness and great mental irritability present. He would not pursue any extended conversation, he would simply reply to simple questions, and when he did reply there was some thickening of the tongue present in these first few weeks. There was a condition of the pulse that was marked, beginning at about 60 to 65, slowing down to from 44 to 48, and remaining there for some weeks. He had subnormal temperature, ranging from 97° to 97.5°. Then he cleared up to some extent. His pulse improved somewhat in character, and he finally was allowed to sit up, and then—about, I think, it was the 7th or 8th of December—it seemed that a change of surroundings and of air would be a good thing for him and he was taken out in the country, when I lost sight of him till a few days ago; having had no opportunity to see him again. His condition at the time, I considered was due to concussion of the brain. While the exact nature of his trouble was to some extent obscure, still there was some profound injury to the brain as indicated by the pulse, the subnormal temperature, the general depression, the thickening of speech—he could not articulate clearly and distinctly—there was some mental wandering, although that was never a marked feature, as far as my own observation went. He was a

City Elevator B, showing opening 102 feet from dock below, through which J. J. W. was forced by the collapse of a large storage tank, similar to the one on adjacent elevator. Some of the supports of the collapsed tank are still seen on Elevator B.

little bit dreamy, I think, at times, as far as I could make out, and he complained of a great deal of pain about the left shoulder, but there was no external or visible evidences of injury that I could determine.

To the question whether he complained of other pain? Dr. Mickle answered yes, although the left shoulder was the chief seat of pain. Earlier he had pains in the head and pains in the back.

From the third to the sixth week after the injury, his pulse varied between 40 and 48, and his temperature ranged from 97.5° F. to 98° F. He complained of pains shooting through his temples and about the left shoulder; his mental condition cleared up slightly and he seemed to improve somewhat.

On September 11 and again on the 13, 1900, I was asked to make a careful examination and report my findings to the representative of the Maryland Casualty Insurance Co. in Buffalo.

The long continued duration of subnormal pulse and temperature, with apathy, dulness of intellect, dull head pains and a loss of memory of recent events, suggested to my mind the possibility of a chronic form of meningitis following a severe cerebral concussion, and my report was made accordingly.

On December 10, 1900, he was taken to his own home on a small farm near Buffalo.

On April 30, 1901, I was permitted to examine him with Dr. Morse, of Batavia, N. Y., and found some improvement in his mental condition, a higher pulse rate, 60 to 69 per minute, no paralytic condition of the cranial nerves and nothing to indicate any organic cerebral lesion. He still complained of pain in his head and back, difficulty in passing urine, stiff, awkward gait, muscular feebleness; in short, he presented the picture of a premature case of senility.

Not being able to arrive at any satisfactory settlement, the case was tried before the Supreme Court, Judge Henry A. Childs, presiding, and a verdict of $7,500 was awarded the plaintiff.

The defense was ably presented by Hon. George A. Clinton, assisted by Messrs. M. Spratt, and Talbot. The plaintiff was represented by Judge S. E. North, of Batavia, and Drs. James W. Putnam, Herbert Mickle, of Buffalo, and H. A. Morse, of Batavia, testified for the plaintiff. The unanimity of opinion among the physicians, that it was a simple case of concussion of the brain without consequent brain lesion, and that the effect produced was to hasten senility and thereby shorten his period of usefulness and productiveness, was appreciated by the court and led to a speedy termination of the case by the jury.

The case is remarkable from the fact that it is possible that a man could be forced out of an opening and be dashed 102 feet to the ground without receiving fatal injuries, or even the slightest abrasion of the skin. The only explanation which I can offer, is that he was surrounded or immersed in the water which served as a cushion or water-bed, thus breaking the shock and thereby saving the man's life.

Case II.—J. H., aged 38 years; height, 5 feet 9 inches; weight, 150 pounds; occupation, painter. His early life was uneventful; was a steady, temperate man; giving no history of alcoholism or syphilis; was a strong, vigorous man. On July 1, 1901, he suffered from a heat stroke, and one week later suffered from a fainting spell. Three weeks later he had a severe attack which undoubtedly was epileptic in character. Six months later he had 3 or 4 of these epileptic attacks in one day. He, however, attended to his work, never losing a day and was examined and accepted for life insurance the early part of the year, 1902.

On Saturday, May 17, 1902, while leaving a street car, he was suddenly thrown backwards by the abrupt braking of the car, striking his back against the seat. A thirty pound pail of paint which he was carrying was thrown to the floor. He was assisted out of the car to a waiting-room on the street corner and after a few minutes took another car to his home. He complained of pain in his back and an abrasion of the skin was found at the level of the 8th thoracic vertebra. He was unable to go to work the following Monday, and on Tuesday, Dr. Willard was called to attend him. The patient let drop a glass from his left hand on that day and this led to the calling of Dr. Willard, who found weakness of the left arm and left leg. On May 31, two weeks after the injury, I was called and found the following condition. A small abrasion about the size of the palm was still present at the middle of the back, about the height of the 8th thoracic vertebra. There was no especial pain or tenderness over this spot. The head offered nothing abnormal. Both sides of the face were symmetrical, the tongue showed no deviation or protrusion. There was marked weakness of the left arm and leg with exaggerated tendon reflexes. Sensation was not impaired. He suffered somewhat with headache, but otherwise was free from pain.

I was inclined to regard the weakness of the arm and leg as of functional origin, because of the non-participation of the face and tongue, being an incomplete hemiplegia, and also because it seemed almost improbable that a direct trauma to the spine could produce an organic lesion of the brain. On the other hand, I could not clearly associate an exaggerated patellar reflex and a true ankle clonus with a functional paralysis, and my diagnosis of the case was left open for future development.

Leaving the city for a few days the patient was attended by Dr. S. A. Dunham, who offers the following information: "I was called on June 4, 1902, and found him suffering with a severe frontal headache, drowsiness, his mind dulled, furred tongue, and an incomplete hemiplegia, having more control over the left leg than over the left arm. He continued to grow rapidly worse, the paresis became more marked, incontinence of urine and feces appeared and his mental faculties were growing duller. He was drowsy and listless most of the time, but when roused up would answer questions without any defect of speech.

"He was removed to the Riverside Hospital on June 18, 1902. His pulse while at hospital ranged from 52 to 58; his temperature varied from 96 3-5°F. to 98 4-5°F. He was always confused mentally, desired to go home, became obstinate, and had one epileptic seizure."

On July 1, he was removed to his home where he grew rapidly weaker, physically as well as mentally. The paresis of the left arm and leg became more pronounced and he gradually fell into a condition of stupor bordering on coma. He had three epileptic seizures while at home.

On July 15, he entered the General Hospital in a comatose condition and at noon on July 16, he suddenly expired. The autopsy made the following day by Dr. H. U. Williams showed the following condition:

Large, not very muscular man; rigor mortis, not marked except jaw. Left upper chest, three large hemorrhagic spots, probably hypodermic marks. Lividity, usual, with small darker areas not marked. Irregular pigmented (light brown) area 3 inches in diameter in lower dorsal region.

While removing skull cap large amount of venous blood escaped. No thrombus in superior longitudinal sinus. Dura not remarkable. Left ventricle, unusual amount of fluid. Nothing found on left side in cortex or basal ganglia. Cerebellum not remarkable. In cutting through right hemisphere find cavity containing blood which comes near surface of brain in median line near fissure of Rolando. Cerebral substance for $1\frac{1}{2}$ inches is discolored yellow to yellow-brown, and is softened. Hemorrhage extends within $\frac{1}{4}$ in. of corpus striatum and optic thalamus, but does not enter either. Hemorrhage lies mostly in centum ovale, coming just to the gray matter of the cortex at fissure of Rolando. Its size is about that of a large walnut, not including yellow area around it. The long axis is perpendicular; contents are clotted blood. About $\frac{3}{4}$ in. in front of it is a cavity, clean cut, thin transparent walls, containing clear fluid; slightly lobulated; cavity about size of a cherry. Diaphragm is at the height of the 5th space on the right side and 6th rib left side.

No fluid in pleural cavities. Left lung free, crepitates well, not remarkable. Right lung adherent slightly at apex, slightly congested posteriorly; irregular area of collapse posteriorly. Crepitates well. Bronchial tubes contain small amount of mucus. Pericardium contains about a teaspoonful of fluid. Heart good size. Pulmonary semilunar valves normal.

Tricuspid normal. Mitral normal. Aortic valves normal. Wall of ventricle in good condition. Aorta normal, no sclerosis. Openings of coronary arteries not sclerosed. Heart weight, $11\frac{1}{2}$ oz. Peritoneum normal. Spleen small, normal consistency, dark red, Malpighian bodies not distinct, trabeculæ distinct. Left kidney weight, 6 oz., large, a little dark, normal consistency, capsule comes off easily, cortex good breadth, medullary rays distinct; ureter normal. Right kidney same as left. Weight, $5\frac{1}{2}$ ozs. Bladder contains 2 or 3 ozs. of amber colored, slightly turbid urine.

Gall-bladder contains no calculi; 1 oz. green bile. Liver good size, a little dark, firm, large amount of venous blood runs from veins on section, dark brown, normal. Pancreas is of good size, pink and normal. Stomach normal, not remarkable except considerable mucus adherent to walls. Ileum normal. Appendix normal.

Anatomical diagnosis—Cortical cerebral hemorrhage right hemisphere; not very recent. Cyst same hemisphere anterior to last.

Microscopical examination of the brain substance between the cyst and the hemorrhage revealed no breaking down of brain tissue, and no aneurysmal condition of the end arteries. The arteries in the hemorrhagic area were not examined. There was no atheromatous condition of the aorta, or of the vessels in the circle of Willis; no renal involvement or valvular disease of the heart, and it was therefore assumed that there was no distinct atheroma of the cerebral vessels present.

The case was stubbornly fought before Judge D. J. Kenefick, of Buffalo; Mr. Clarence Bushnell, appearing for the plaintiff and Mr. Thomas Penney, for the defendant. After an exhaustive presentation of facts by experts on both sides, the jury awarded the plaintiff a verdict of $7,000.00.

The case was essentially one for a jury to determine, as there was a tenable position for both sides to maintain. The finding of the cyst cephalad of the hemorrhage was unexpected and was unaccompanied by any focal symptoms. Its origin or date of development was impossible to determine, except that in all probability it antedated the hemorrhage for a long time. The onset of the paralysis of the left arm and leg was gradual, owing to the slowly increasing hemorrhage into the brain substance at the junction of the arm and leg centers and explained why the lower segment of the left face and tongue were not involved. The hemorrhage was subcortical and consequently did not produce any focal seizures. The paralytic condition closely resembled a functional or hysterical hemiplegia and without the autopsy the true condition of the brain involvement would have been unknown. This case makes imperative the complete investigation of ante- and post-mortem conditions, where litigation is apt to follow, or where liability is even remotely involved.

479 Delaware Avenue.

## Figures and Tables

**Figure f1:**